# Modification of Platelet Count on the Association between Homocysteine and Blood Pressure: A Moderation Analysis in Chinese Hypertensive Patients

**DOI:** 10.1155/2020/5983574

**Published:** 2020-02-14

**Authors:** Jianan Zhang, Jing Li, Shi Chen, Linglin Gao, Xiaoluan Yan, Mingzhi Zhang, Jia Yu, Fenchun Wang, Hao Peng

**Affiliations:** ^1^Department of Epidemiology, School of Public Health and Jiangsu Key Laboratory of Preventive and Translational Medicine for Geriatric Diseases, Medical College of Soochow University, Suzhou, China; ^2^Center for Disease Prevention and Control of Taicang City, Suzhou, China; ^3^Department of Cardiology, Suzhou Municipal Hospital Affiliated to Nanjing Medical University, Suzhou, China

## Abstract

**Background:**

Platelet consumption followed by homocysteine-induced endothelial injury suggests a crosstalk between platelet activation and homocysteine on hypertension. Platelet count has been found to modify the effect of folic acid on vascular health. However, whether platelet count could modify the contribution of homocysteine to blood pressure (BP) remains unclear.

**Methods:**

Leveraging a community-based cross-sectional survey in 30,369 Chinese hypertensive patients (mean age 62 years, 52% female), we examined the moderation of platelet count on the association between serum homocysteine and BP by constructing hierarchical multiple regression models, adjusting for conventional risk factors. If adding the interaction term of homocysteine and platelet count could explain more variance in BP and the interaction is significant, then we believe that moderation is occurring.

**Results:**

The association between serum homocysteine and diastolic BP was significantly stronger (*β* = 0.092 vs. 0.035, *P* = 0.004) in participants with low platelet count (<210 × 10^9^/L) than in those with high platelet count (≥210 × 10^9^/L). Adding the interaction term of homocysteine and platelet count additionally explained 0.05% of the variance in diastolic BP (*P* = 0.004) in participants with low platelet count (<210 × 10^9^/L) than in those with high platelet count (≥210 × 10^9^/L). Adding the interaction term of homocysteine and platelet count additionally explained 0.05% of the variance in diastolic BP (*β* = 0.092 vs. 0.035, *P* = 0.004) in participants with low platelet count (<210 × 10^9^/L) than in those with high platelet count (≥210 × 10^9^/L). Adding the interaction term of homocysteine and platelet count additionally explained 0.05% of the variance in diastolic BP (

**Conclusions:**

The association between homocysteine and BP was significantly stronger in participants with low *vs*. high platelet count and was partially moderated by platelet count. These results indicate that platelet count may be useful in the identification of individuals who are most beneficial to reducing-homocysteine treatments but this usefullness still needs further investigation.

## 1. Introduction

Essential hypertension is highly prevalent all over the world and a leading modifiable risk factor for cardiovascular disease (CVD) [1, 2]. Better understanding the underlined mechanisms of hypertension is likely to improve prevention and management of this debilitating disorder and reduce related disease burden. Homocysteine (Hcy) has been involved in the pathogenesis of hypertension by producing endothelial injury [3, 4], increasing oxidative stress [5], stimulating the proliferation of vascular smooth muscle cells (VSMCs) [6], and altering the elastic properties of the vascular wall [7]. Elevated Hcy and its related genetic variants have been associated with blood pressure [8–12], hypertension [13–15], and related vascular complications, e.g., atherosclerosis [16, 17], coronary heart disease [18, 19], and stroke [20, 21], in humans. In the process of Hcy-induced hypertension, the endothelial injury resulting from Hcy promotes platelet consumption and adherence which stimulates the proliferation of VSMCs through releasing mitogenic factors [22], thereby contributing to atherosclerosis, arterial stiffness, and high blood pressure [23, 24] Furthermore, platelet activation has been associated with Hcy [25] but also hypertension [26] in population studies. As such, platelet response may play a potential role in enhancing the contribution of Hcy to the risk and severity of hypertension. In support of this hypothesis, a recent trial found that platelet count interacted with Hcy and modified the effect of folic acid on risk of stroke [27]. To date, however, the moderation or interaction between Hcy and platelet count on blood pressure has been poorly studied but deciphering their crosstalk is likely to provide novel insights into mechanisms of hypertension and may also identify novel therapeutic targets for this debilitating disorder and related conditions. The aim of this study was to examine the moderation and interaction of platelet count on the association between homocysteine and blood pressure in more than 30 thousand Chinese adults with essential hypertension.

## 2. Methods

### 2.1. Participants

Hyperhomocysteinemia (HHcy) has been considered extremely and highly prevalent in Chinese population in the last decades due to lack of mandatory folic acid fortification of grain products, insufficient consumption of folate-containing foods, a low rate of folic acid supplementation, and a high prevalence of the methylenetetrahydrofolate reductase (*MTHFR*) C677T gene mutation (25% in Chinese vs. 10% to 12% in the general U.S. population) [28] After decades of efforts, e.g., health education and dietary promotion, the prevalence of HHcy is supposed to be declined in this population, hypertensive patients in particular. To estimate the prevalence of HHcy among Chinese hypertensive patients, we conducted a community-based cross-sectional survey in 21 communities residing in Taicang, a traditional but economically developed county in China. There is an effective system of registry and management of chronic diseases, including hypertension, diabetes, and CVD, covering all of the adult residents in this county. More than 70,000 living hypertensive patients were registered in this system by the end of 2016. Of them, 30,379 patients agreed to participate in our cross-sectional clinical examination operated in 2017 and gave written informed consent. After excluding participants with missing data on either serum Hcy or platelet count (*N* = 100), a total of 30,369 hypertensive patients were finally included in the current analysis. Hypertension defined as SBP ≥ 140 mmHg and/or DBP ≥ 90 mmHg or use of antihypertensive medications in the last 2 weeks was used in our study [29].

### 2.2. Assessment of Homocysteine

Overnight fasting venous blood samples were collected for each participant and shipped to the core laboratory of the Center for Disease Prevention and Control of Taicang within four hours after venipuncture. All laboratory tests were performed at this laboratory on the same day. Serum total Hcy was determined by chemistry enzymatic assay (Axis-Shield Diagnostics Ltd., Dundee, UK) on the Cobas c501 analyzer (Roche Diagnostics, Indianapolis, IN). The intra- and interassay coefficients of variation were less than 7.5% and 4.5%, respectively.

### 2.3. Measurement of Blood Pressure

Blood pressure was measured three times by trained staff using a standard mercury sphygmomanometer and a cuff of appropriate size according to a standard protocol [29], after the participants had been resting for at least 5 min in a relaxed, sitting position. The first and fifth Korotkoff sounds were recorded as systolic blood pressure (SBP) and diastolic blood pressure (DBP), respectively. The mean of the three measurements was used in statistical analyses.

### 2.4. Measurement of Other Risk Factors

Blood cell analysis, including platelet count, plateletcrit, platelet distribution width, and mean platelet volume, was obtained using a BC-3200 hematology analyzer (Mindray Medical, Shenzhen, China). Fasting glucose, blood lipids including total cholesterol, triglycerides, high-density lipoprotein cholesterol (HDL-C), and low-density lipoprotein cholesterol (LDL-C), and creatinine were measured by standard laboratory methods [30]. Diabetes was defined as fasting glucose over 7.0 mmol/L and/or receiving hypoglycemic medications in the last 2 weeks [31]. Body weight (kg) and height (cm) were measured when participants wore light clothes and no shoes by trained staff. Body mass index (BMI) was calculated by dividing weight in kilograms by the square of height in meters (kg/m^2^).

### 2.5. Statistical Analysis

Given the extreme sample of only hypertensive patients included in our study, *z*-transformation was applied to standardize the distribution of SBP and DBP with a mean of 0 and a standard deviation of 1. The generated *z*-scores were used in all downstream analyses. To examine whether platelet count modifies the association between Hcy and blood pressure, we first constructed a robust linear regression model in which z-transformed SBP/DBP was the dependent variable and log-transformed Hcy (log-Hcy) was the independent variable, adjusting for age, sex, receiving antihypertensive medication (y/*n*), fasting glucose, LDL-C, HDL-C, and creatinine in participants with low and high platelet count (less than vs. over 210 × 10_9_/L), due to that platelet count less than 210 × 10_9_/L has been identified to modify the efficacy of folic acid in preventing stroke in Chinese hypertensive patients [27]. The robust regression model was used here to account for the influence of extreme values on the model fitting. The modification of platelet count levels on the association between Hcy and blood pressure was tested by comparing the regression coefficients of the linear models fitted in participants with low *vs*. high platelet count. To further examine whether and to what extent does platelet count moderate the association between Hcy and blood pressure, we constructed the following hierarchical multiple regression models:

- Model 1: the regression of blood pressure on log-Hcy and platelet count, adjusting for covariates listed above
- Model 2: add the interaction term of log-Hcy × platelet count as a predictor to model 1

To facilitate comparison, all variables were centered to a distribution with a mean of 0 and a standard variation of 1. If the *R*^2^ of Model 2 is significantly improved compared with Model 1 and the interaction effect is significant, then we believe that moderation is occurring. Moderation analysis was performed using *R* package “medmod” [32].

### 2.6. Sensitivity Analysis

To examine whether medications affect our results, we repeated the analysis after excluding participants receiving antihypertensive or antidiabetic medications. We additionally examined the moderation of other markers of platelet consumption including plateletcrit, platelet distribution width, and mean platelet volume on the association between Hcy and blood pressure. All statistical analyses were conducted using SAS statistical software (version 9.4; Cary, North Carolina, USA) unless otherwise noted. A two-tailed *P* value of less than 0.05 was considered statistically significant.

## 3. Results

This study included 30,369 hypertensive patients (mean age 62 years, 52% female) including 4,160 (14%) patients not receiving antihypertensive drugs. Among these, 61%, 43%, and 22% individuals suffered HHcy defined as a serum Hcy level over 10, 12, and 15 *μ*mol/L, respectively.  Table 1 presents the clinical characteristics of these hypertensive patients according to platelet count. Participants with low platelet count were more likely to be elder, male, and have lower levels of SBP, DBP, BMI, blood lipids, and serum Hcy, but a higher level of creatinine than those with high platelet count (all *P* < 0.001). Higher levels of platelet consumption as indicated by higher MPV and PDW and lower PCT occurred in participants with low platelet count compared with those with high platelet count (all *P* < 0.05).

### 3.1. Association between Hcy and Blood Pressure according to Platelet Count

Table 2 shows the associations of serum Hcy with SBP and DBP in hypertensive patients with low *vs*. high platelet count, independent of age, sex, BMI, fasting glucose, lipids, kidney function, and antihypertensive medication. In participants with low platelet count, higher serum Hcy was significantly associated with higher SBP (bottom-line significant with *β* = 0.026, *P*=0.079) and DBP (*β* = 0.092, *P* < 0.001), whereas these associations were not significant in participants with high platelet count. And the magnitudes of the associations of serum Hcy with SBP (*P*=0.017) and DBP (*P*=0.004) in hypertensive patients with low platelet count were much stronger than that in those with high platelet count. These results indicated a possible modification of platelet count on the association between serum Hcy and blood pressure.

### 3.2. Moderation of Platelet Count on the Association between Hcy and Blood Pressure

The results of moderation analysis further demonstrated the modification of platelet count on the association between Hcy and DBP. As shown in Table 3, after further adjusting for the interaction term of Hcy and platelet count, the magnitude of the association between serum Hcy and DBP remained unchanged and significant but the *R*^2^ of the regression model significantly increased by 0.0005 (*P*=0.0001). Furthermore, the interaction between Hcy and platelet count was significantly associated with DBP (*β* = −0.021, *P* < 0.001). These results indicated that the association between Hcy and DBP may be partially moderated by platelet count and seemed to be stronger in participants with low platelet count. We failed to identify statistically but bottom-line significant moderation of platelet count on the association between Hcy and SBP (*β* = −0.011, *P*=0.057).

### 3.3. Results of Sensitivity Analysis

After excluding participants receiving antihypertensive drugs (*N* = 26,209), the association between Hcy and blood pressure was significant in patients with low platelet count but not significantly stronger than that in patients with high platelet count (Supplementary [Supplementary-material supplementary-material-1]). Although we failed to observe a statistically significant moderation of platelet count on the association between Hcy and blood pressure in this small subsample of hypertensive patients, the inconsistent results in patients with low *vs*. high platelet count may support our findings in total participants. Excluding participants receiving antidiabetic medications did not change our results (Supplementary [Supplementary-material supplementary-material-1]). In addition to platelet count, plateletcrit also significantly moderated the association between Hcy and blood pressure (all *P* < 0.05, Supplementary [Supplementary-material supplementary-material-1]). We did not find significant moderation of platelet distribution width or volume on the association between Hcy and blood pressure (Supplementary Tables [Supplementary-material supplementary-material-1] and [Supplementary-material supplementary-material-1]).

## 4. Discussion

A recent clinical trial including 10,789 Chinese hypertensive adults found that platelet count modified the effect of folic acid supplementation on the risk of incident stroke [27]. This secondary analysis of China Stroke Primary Prevention Trial (CSPPT) introduced for the first time a probable moderating effect of platelet count on the contribution of Hcy to risks of stroke and other vascular disorders, such as hypertension, but it may be prone to uncover a false-positive finding. In order to replicate this finding of interest, our study examined the moderation of platelet count on the association between Hcy and blood pressure, leveraging a large sample of hypertensive patients with available data on Hcy. The results showed that the association between Hcy and blood pressure, DBP in particular, was partially moderated by platelet count and was significantly stronger in participants with low platelet count than that in those with high platelet count, regardless of antihypertensive medications and metabolic risk factors. Platelet consumption may be involved in the progression of hypertension through mechanisms beyond metabolic risk factors.

In line with our study, the identified association between Hcy and blood pressure was also demonstrated by many previous studies. For example, basic experiments found that injection of Hcy resulted in blood pressure elevation in rats [33]. Genome-wide association studies revealed that several variants in the *MTHFR*, a gene regulating Hcy synthesis [34], were associated with blood pressure in humans, regardless of hypertensive status and antihypertensive medications [8, 9, 35]. The positive association between circulating Hcy and blood pressure has also been found in diverse populations by many large-scale epidemiological studies [36], including the Third National Health and Nutrition Examination Survey (NHANES) [37]. The association between Hcy and blood pressure has been mostly studied in general populations comprising normal and hypertensive individuals, and the role of Hcy in the progression of hypertension is not well studied. Our study included only hypertensive individuals and found a significant association between Hcy and blood pressure in this population and therefore may provide an initial evidence for the contributing role of Hcy in the progression of hypertension.

In addition to the association between Hcy and blood pressure, the other important goal of our study is to examine the moderation effect of platelet count on this association. In line with the results from the CSPPT [27], we found that the association between Hcy and blood pressure was significantly moderated by platelet count, where this association was significantly stronger for participants with low platelet count. Although the underlined mechanisms are not clear, some probable mechanisms shared by Hcy and platelet involved in vascular health may explain, at least partly, the moderating effect of platelet count on the association between Hcy and blood pressure. As an illustration, as a fundamental pathogenesis of hypertension, endothelial dysfunction could be resulted by Hcy [38] and can stimulate aggregation and adherence of platelet [39]. Platelet consumption and activation consequently stimulate the proliferation of VSMCs by releasing mitogenic factors [22]. Further, Hcy per se could also induce the proliferation of VSMCs 6 probably through interaction with platelet, indicated by an Hcy-dependent expression of the platelet-derived growth factor [40], thereby contributing to atherosclerosis, arterial stiffness, and high blood pressure [23, 24]. Together with these basic studies, our findings may suggest that the contribution of Hcy to atherosclerosis and the progression of hypertension may be boosted in individuals with a status of high active platelet consumption. In further support of this speculation, we found that higher Hcy (after log-transformation) was significantly associated with higher levels of platelet count (*β* = 1.28, *P* = 0.045), mean platelet volume (*β* = 0.059, *P* = 0.005), and plateletcrit (*β* = 0.002, *P* = 0.002) and a lower level of platelet distribution width (*β* = −0.029, *P* = 0.013) in our study. We also found that plateletcrit, a complementary analysis of platelet count and measures the percentage of platelets in the blood [41], significantly moderated the association between Hcy and blood pressure in our study. The more active is platelet consumed, the fewer platelets are in the circulation. Although we failed to identify a statistically significant moderation for the other two markers of platelet consumption activation, platelet distribution width and mean platelet volume [42, 43], the identified statistically significant moderation of platelet count and plateletcrit on the association between Hcy and blood pressure, together with prior consistent findings, may shed light on a biological moderation effect of platelet consumption on modifying vascular toxicity of Hcy. The vascular toxicity of Hcy, indicated by a higher blood pressure here, seems relatively greater in individuals who do not have reduced platelet production. Of note, we found that the magnitude of the association between Hcy and DBP seemed higher than that between Hcy and SBP. This phenomenon is also observed in prior population studies [44]. Although some mechanisms through which Hcy leads to DBP dysfunction have been indicated, e.g., reduced the production of vasodilators such as endogenous nitric oxide [45], reduced arterial wall compliance through impairing vascular matrix metalloproteinase activity [46], and increased endothelial-myocyte uncoupling [47], the mechanisms underlining this interesting phenomenon are still not very clear and warranted further investigation because DBP was considered associated with blood flow in the brain.

This study reserves the first to examine the moderation of platelet count on the association between Hcy and blood pressure in populations. The strengths of our study included the large sample size, comprehensive assessment, and adjustment for multiple confounding factors includingmetabolic factors, stratification analysis by using antihypertensive medications to eliminate its bias, and the careful moderation analysis by constructing hierarchical multiple regression models. Some limitations of our study also should be acknowledged. First, the cross-sectional study design prevents us from exploring the causal or temporal relationship between Hcy, platelet count, and blood pressure elevation. Second, only Chinese hypertensive adults were included in our study. Given the higher prevalence of HHcy in Chinese than European ancestry populations [28], the generalizability of our findings to other ethnic groups, nonhypertensive adults, or younger populations is concerned. Third, to facilitate data interpretation, we used the same cutoff value of platelet count as used in the CSPPT [27]. In fact, it is still unclear about the reference level of platelet count that could be used in clinical settings to identify individuals who are sensitive to Hcy or folic acid supplementation. However, our study may suggest that platelet count possesses a potential to identify individuals who are the most appropriate for the treatment of folic acid or vitamin B12. Fourth, we did not obtain data on organ damage of hypertension, e.g., CVD events and chronic kidney disease, although serum creatinine was adjusted for in our statistical analysis. Whether and to what extent these organic dysfunctions influence our results is not clear. Fifth, although blood pressure was measured three times, the level of blood pressure under analysis was obtained on only 1 occasion. Inaccuracy may still exist and may affect our data interpretation. Sixth, we did not obtain data on the use of B12 or folate supplements which directly influence Hcy levels.

## Figures and Tables

**Table 1:** Clinical characteristics of study participants according to platelet count.

Characteristics	Low platelet count (<210 × 10^9^/L)	High platelet count (≥210 × 10^9^/L)	*P*
No. of participants	21,980	8,389	
Age (years)	63.3 ± 7.1	61.7 ± 7.2	<0.001
Sex, men (%)	11,087 (50.44)	3,516 (41.91)	<0.001
Antihypertensive medication, *n* (%)	18,897 (85.97)	7,312 (87.16)	0.007
Body mass index (kg/m^2)^	24.71 ± 3.36	25.08 ± 3.31	<0.001
Fasting glucose (mmol/L)	5.92 ± 1.48	5.94 ± 1.46	0.252
Total cholesterol (mmol/L)	4.82 ± 1.01	5.10 ± 1.04	<0.001
Triglycerides (mmol/L)	1.80 ± 1.40	1.96 ± 1.43	<0.001
LDL-C (mmol/L)	2.78 ± 0.81	3.06 ± 0.84	<0.001
HDL-C (mmol/L)	1.49 ± 0.48	1.47 ± 0.45	<0.001
Creatinine (mmol/L)	73.37 ± 36.96	69.57 ± 30.47	<0.001
Systolic blood pressure (mmHg)	143.8 ± 17.6	144.6 ± 17.5	<0.001
Diastolic blood pressure (mmHg)	83.0 ± 10.5	84.0 ± 10.7	<0.001
Log-Hcy	2.40 ± 0.49	2.41 ± 0.48	0.037
Platelet consumption			
Mean platelet volume (fL)	10.10 ± 1.75	8.87 ± 1.45	<0.001
Platelet crit (%)	0.1559 ± 0.0350	0.2192 ± 0.0450	<0.001
Platelet distribution width (%)	16.36 ± 0.93	15.90 ± 1.08	<0.001

All results are expressed in mean ± SD unless otherwise noted. LDL-C: low-density lipoprotein cholesterol; HDL-C: high-density lipoprotein cholesterol; Log-Hcy: log-transformed homocysteine.

**Table 2:** The association between serum homocysteine and blood pressure according to platelet count.

Subgroups	*z*-transformed SBP			*z*-transformed DBP		
	*β* ^*∗*^ (SE)	*P* ^*∗*^	*P* ^‡^ for difference in *β*	*β* ^‡^ (SE)	*P* ^‡^	*P* ^‡^ for difference in *β*
Total participants	0.017 (0.013)	0.181	—	0.078 (0.012)	<0.001	—
Subgroup by quartiles of platelet count						
Q1 (<146 × ×10^9^/L)	−0.010 (0.025)	0.683	0.249	0.108 (0.024)	<0.001	0.003
Q2 (146–178 × 10^9^/lL)	0.053 (0.026)	0.044		0.098 (0.025)	<0.001	
Q3 (179–213 × 10^9^/lL)	0.044 (0.026)	0.091		0.058 (0.025)	0.019	
Q4 (≥214 × 10^9^/L)	−0.027 (0.026)	0.297		0.039 (0.025)	0.119	
Subgroup by platelet count referring to CSPPT						
Platelet count <210 × 10^9^/L	0.026 (0.015)	0.079	0.017	0.092 (0.014)	<0.001	0.004
Platelet count ≥210 × 10^9^/L	−0.017 (0.025)	0.502		0.035 (0.024)	0.144	

^*∗*^The increase of *z*-transformed SBP (*β*) per unit increment of log-transformed homocysteine and its significance test (*P*); ^‡^the increase of *z*-transformed DBP (*β*) per unit increment of log-transformed homocysteine and its significance test (*P*); ^‡^the significance test of the difference in the regression coefficients between the two subgroups.

**Table 3:** Moderation of platelet count on the association between serum homocysteine and blood pressure.

Independent variables	Model 1			Model 2		
	*β* (SE)	*P*	*R* ^2^	*β* (SE)	*P*	*R* ^2^
*z-transformed SBP*						
*z*-transformed log-Hcy	0.007 (0.006)	0.240	0.0478	0.007 (0.006)	0.271	0.0479
*z*-transformed platelet count	0.017 (0.006)	0.004		0.017 (0.006)	0.005	
Interaction term	—	—		−0.011 (0.006)	0.057	
Moderation tests	Δ*R*^2^ = 0.0001, *F* = 2.9585, *P*=0.085					
*z-transformed DBP*						
*z*-transformed log-Hcy	0.036 (0.006)	<0.001	0.0975	0.035 (0.006)	<0.001	0.0980
*z*-transformed platelet count	0.019 (0.006)	0.001		0.019 (0.006)	0.001	
Interaction term	—	—		−0.021 (0.005)	<0.001	
Moderation tests	Δ*R*^2^ = 0.0005, *F* = 14.734, *P*=0.0001					

Log-Hcy: log-transformed homocysteine; model 1: the regression of blood pressure on z‐transformed log-Hcy and z‐transformed platelet count, adjusting for age, sex, body mass index, fasting glucose, lipids, and creatinine; model 2: further adjusting for the interaction term of z‐transformed log‐Hcy and z‐transformed platelet count.

## Data Availability

All information analyzed is deidentified, and the anonymous data will be shared by a request to the corresponding author at penghao@suda.edu.cn.

## Additional Points

*Perspectives*. The moderation effect of platelet count on the contribution of Hcy to vascular disorders suggested by the CSPPT [27] was successfully demonstrated by our study in a large sample of Chinese hypertensive adults. The association between serum Hcy and blood pressure was significantly stronger in participants with low *vs*. high platelet count and was partially moderated by platelet count. These results indicate that platelet count may be useful in the identification of individuals who are most beneficial to reducing-homocysteine treatments but this potential usefullness still needs further investigation.

## Supplementary Materials

Supplementary Materials To further examine the robustness of our results, we performed a series of sensitivity analyses. Specifically, to examine whether antihypertensive medications affect our results, we repeated the analyses in participants receiving antihypertensive medications or not. As shown in Supplementary Table S1, after excluding participants receiving antihypertensive drugs (*N* = 26,209), the association between Hcy and blood pressure was significant in patients with low platelet count but not significantly stronger than that in patients with high platelet count. Although we failed to observe a statistically significant moderation of platelet count on the association between Hcy and blood pressure in this small subsample of hypertensive patients, the inconsistent results in patients with low vs. high platelet count may support our findings in total participants. To examine whether receiving antidiabetic medications affects our results, we repeated the analyses in participants receiving antidiabetic medications or not. As shown in Supplementary Table S2, excluding participants receiving antidiabetic medications did not change our results. The association between Hcy and blood pressure was significantly stronger in participants with low platelet counts. To examine whether other index of platelet consumption could modify the association between Hcy and blood pressure, we additionally examined the association between Hcy and blood pressure in subgroups by plateletcrit (Supplementary Table S3), platelet distribution width (Supplementary Table S4), and mean platelet volume (Supplementary Table S5). We found that plateletcrit also significantly moderated the association between Hcy and blood pressure (all *P* < 0.05). We did not find significant moderation of platelet distribution width or volume on the association between Hcy and blood pressure.
