# Supplementary material for: Modification of Platelet Count on the Association between Homocysteine and Blood Pressure: A Moderation Analysis in Chinese Hypertensive Patients
Source: Int J Hypertens. 2020 Feb 14;2020:5983574. doi: 10.1155/2020/5983574 (PMC7048938; doi:10.1155/2020/5983574)
Supplement: Supplementary Materials — To further examine the robustness of our results, we performed a series of sensitivity analyses. Specifically, to examine whether antihypertensive medications affect our results, we repeated the analyses in participants receiving antihypertensive medications or not. As shown in Supplementary Table S1, after excluding participants receiving antihypertensive drugs (N = 26,209), the association between Hcy and blood pressure was significant in patients with low platelet count but not significantly stronger than that in patients with high platelet count. Although we failed to observe a statistically significant moderation of platelet count on the association between Hcy and blood pressure in this small subsample of hypertensive patients, the inconsistent results in patients with low vs. high platelet count may support our findings in total participants. To examine whether receiving antidiabetic medications affects our results, we repeated the analyses in participants receiving antidiabetic medications or not. As shown in Supplementary Table S2, excluding participants receiving antidiabetic medications did not change our results. The association between Hcy and blood pressure was significantly stronger in participants with low platelet counts. To examine whether other index of platelet consumption could modify the association between Hcy and blood pressure, we additionally examined the association between Hcy and blood pressure in subgroups by plateletcrit (Supplementary Table S3), platelet distribution width (Supplementary Table S4), and mean platelet volume (Supplementary Table S5). We found that plateletcrit also significantly moderated the association between Hcy and blood pressure (all P < 0.05). We did not find significant moderation of platelet distribution width or volume on the association between Hcy and blood pressure. [file 5983574.f1.docx]

**Supplementary Data**

To further examine whether antihypertensive medications affect our results, we repeated the analyses in participants receiving antihypertensive medications or not. The results showed that after excluding participants receiving antihypertensive drugs (N=26,209), the associations between Hcy and blood pressure was significant in patients with low platelet count but not significantly stronger than that in patients with high platelet count. Although we failed to observe a statistically significant moderation of platelet count on the association between Hcy and blood pressure in this small subsample of hypertensive patients, the inconsistent results in patients with low vs. high platelet count may support our findings in total participants.

| **Supplementary Table S1**. The association between serum homocysteine and blood pressure according to platelet count in participants receiving antihypertensive medication or not | | | | | | | |
| --- | --- | --- | --- | --- | --- | --- | --- |
| Subgroups | z-transformed SBP | | |  | z-transformed DBP | | |
|  | β^*^ (SE) | *P*^*^ | *P*^‡^ for difference in β |  | β^†^ (SE) | *P*^†^ | *P*^‡^ for difference in β |
| Not receiving antihypertensive mediation (N=4,160) | | | | | | | |
| Platelet count <210×10^9^/L | 0.245 (0.044) | <0.001 | 0.393 |  | 0.160 (0.041) | <0.001 | 0.381 |
| Platelet count ≥210×10^9^/L | 0.079 (0.081) | 0.333 |  |  | 0.099 (0.077) | 0.201 |  |
| receiving antihypertensive mediation (N=26,209) | | | | | | | |
| Platelet count <210×10^9^/L | -0.014 (0.016) | 0.397 | 0.203 |  | 0.078 (0.015) | <0.001 | 0.021 |
| Platelet count ≥210×10^9^/L | -0.049 (0.026) | 0.064 |  |  | 0.020 (0.026) | 0.430 |  |
| ^*^the increase of z-transformed SBP (β) per unit increment of log-transformed homocysteine and its significance test (*P*);  ^†^the increase of z-transformed DBP (β) per unit increment of log-transformed homocysteine and its significance test (*P*);  ^‡^the significance test of the difference in the regression coefficients between the two subgroups. | | | | | | | |

Excluding participant receiving antidiabetic medications did not change our results.

| **Supplementary Table S2**. The association between serum homocysteine and blood pressure according to platelet count in participants receiving antidiabetic medication or not | | | | | | | |
| --- | --- | --- | --- | --- | --- | --- | --- |
| Subgroups | z-transformed SBP | | |  | z-transformed DBP | | |
|  | β^*^ (SE) | *P*^*^ | *P*^‡^ for difference in β |  | β^†^ (SE) | *P*^†^ | *P*^‡^ for difference in β |
| Not receiving antidiabetic mediation (N=26,314) | | | | | | | |
| Platelet count <210×10^9^/L | 0.028 (0.016) | 0.081 | 0.012 |  | 0.097 (0.015) | <0.001 | 0.006 |
| Platelet count ≥210×10^9^/L | -0.027 (0.027) | 0.316 |  |  | 0.036 (0.026) | 0.160 |  |
| receiving antidiabetic mediation (N=4,055) | | | | | | | |
| Platelet count <210×10^9^/L | 0.017 (0.048) | 0.721 | 0.963 |  | 0.065 (0.045) | 0.142 | 0.609 |
| Platelet count ≥210×10^9^/L | 0.022 (0.076) | 0.770 |  |  | 0.042 (0.072) | 0.563 |  |
| ^*^the increase of z-transformed SBP (β) per unit increment of log-transformed homocysteine and its significance test (*P*);  ^†^the increase of z-transformed DBP (β) per unit increment of log-transformed homocysteine and its significance test (*P*);  ^‡^the significance test of the difference in the regression coefficients between the two subgroups. | | | | | | | |

In addition to platelet count, plateletcrit also significantly moderated the association between Hcy and blood pressure.

| **Supplementary Table S3**. Moderation of platelet crit on the association between serum homocysteine and blood pressure | | | | | | | |
| --- | --- | --- | --- | --- | --- | --- | --- |
| Independent variables | Model 1 | | |  | Model 2 | | |
|  | β (SE) | *P* | R^2^ |  | β (SE) | *P* | R^2^ |
| **z-transformed SBP** | | | | | | | |
| z-transformed log-Hcy | 0.008(0.006) | 0.232 | 0.0395 |  | 0.006(0.006) | 0.348 | 0.0398 |
| z-transformed platelet crit | -0.002(0.006) | 0.726 |  |  | -0.001(0.006) | 0.845 |  |
| Interaction term | - | - |  |  | -0.018(0.006) | 0.003 |  |
| Moderation tests | ΔR^2^=0.0003, F=6.7196, *P*=0.010 | | | | | | |
| **z-transformed DBP** | | | | | | | |
| z-transformed log-Hcy | 0.041(0.006) | <0.001 | 0.0914 |  | 0.039(0.006) | <0.001 | 0.0918 |
| z-transformed platelet crit | -0.011(0.006) | 0.059 |  |  | -0.01(0.006) | 0.082 |  |
| Interaction term | - | - |  |  | -0.021(0.006) | <0.001 |  |
| Moderation tests | ΔR^2^=0.0004, F=13.9930, *P*=0.0002 | | | | | | |
| Log-Hcy: log-transformed homocysteine;  Model 1: including z-transformed log-Hcy, z-transformed platelet count, age, sex, body mass index. Fasting glucose, lipids, and creatinine as the independent variables;  Model 2: model 1 plus the interaction term of z-transformed log-Hcy and z-transformed platelet count. | | | | | | | |

We did not find significant moderation of platelet distribution width on the association between Hcy and blood pressure.

| **Supplementary Table S4**. Moderation of platelet distribution width on the association between serum homocysteine and blood pressure | | | | | | | |
| --- | --- | --- | --- | --- | --- | --- | --- |
| Independent variables | Model 1 | | |  | Model 2 | | |
|  | β (SE) | *P* | R^2^ |  | β (SE) | *P* | R^2^ |
| **z-transformed SBP** | | | | | | | |
| z-transformed log-Hcy | 0.001(0.006) | 0.826 | 0.0394 |  | 0.001(0.006) | 0.826 | 0.0394 |
| z-transformed PDW | 0.011(0.006) | 0.064 |  |  | 0.011(0.006) | 0.072 |  |
| Interaction term | - | - |  |  | 0.000(0.007) | 0.997 |  |
| Moderation tests | ΔR^2^=0.0000, F=0.0188, *P*=0.891 | | | | | | |
| **z-transformed DBP** | | | | | | | |
| z-transformed log-Hcy | 0.036(0.006) | <0.001 | 0.0904 |  | 0.036(0.006) | <0.001 | 0.0904 |
| z-transformed PDW | 0.049(0.006) | <0.001 |  |  | 0.049(0.006) | <0.001 |  |
| Interaction term | - | - |  |  | -0.001(0.007) | 0.946 |  |
| Moderation tests | ΔR^2^=0.0000, F=0.1451, *P*=0.7033 | | | | | | |
| Log-Hcy: log-transformed homocysteine;  Model 1: including z-transformed log-Hcy, z-transformed platelet count, age, sex, body mass index. Fasting glucose, lipids, and creatinine as the independent variables;  Model 2: model 1 plus the interaction term of z-transformed log-Hcy and z-transformed platelet count. | | | | | | | |

We did not find significant moderation of mean platelet volume on the association between Hcy and blood pressure.

| **Supplementary Table S5**. Moderation of mean platelet volume on the association between serum homocysteine and blood pressure | | | | | | | |
| --- | --- | --- | --- | --- | --- | --- | --- |
| Independent variables | Model 1 | | |  | Model 2 | | |
|  | β (SE) | *P* | R^2^ |  | β (SE) | *P* | R^2^ |
| **z-transformed SBP** | | | | | | | |
| z-transformed log-Hcy | 0.008(0.006) | 0.214 | 0.0405 |  | 0.007(0.006) | 0.240 | 0.0405 |
| z-transformed MPV | -0.033(0.006) | <0.001 |  |  | -0.032(0.006) | <0.001 |  |
| Interaction term | - | - |  |  | -0.007(0.006) | 0.247 |  |
| Moderation tests | ΔR^2^=0.0000, F=0.8834, *P*=0.347 | | | | | | |
| **z-transformed DBP** | | | | | | | |
| z-transformed log-Hcy | 0.040(0.006) | <0.001 | 0.0924 |  | 0.041(0.006) | <0.001 | 0.0925 |
| z-transformed MPV | -0.035(0.006) | <0.001 |  |  | -0.037(0.006) | <0.001 |  |
| Interaction term | - | - |  |  | 0.010(0.006) | 0.104 |  |
| Moderation tests | ΔR^2^=0.0001, F=3.0684, *P*=0.080 | | | | | | |
| Log-Hcy: log-transformed homocysteine;  Model 1: including z-transformed log-Hcy, z-transformed platelet count, age, sex, body mass index. Fasting glucose, lipids, and creatinine as the independent variables;  Model 2: model 1 plus the interaction term of z-transformed log-Hcy and z-transformed platelet count. | | | | | | | |
